# Supplementary material for: Nasal Cytology on 241 Children: From Birth to the First 3 Years of Life and Association with Common Airways Diseases
Source: J Pers Med. 2023 Apr 19;13(4):687. doi: 10.3390/jpm13040687 (PMC10146100; doi:10.3390/jpm13040687)
Supplement: Supplementary file 1 [file jpm-13-00687-s001.zip › jpm-2324822-supplementary.pdf]

Supplemetary Materials

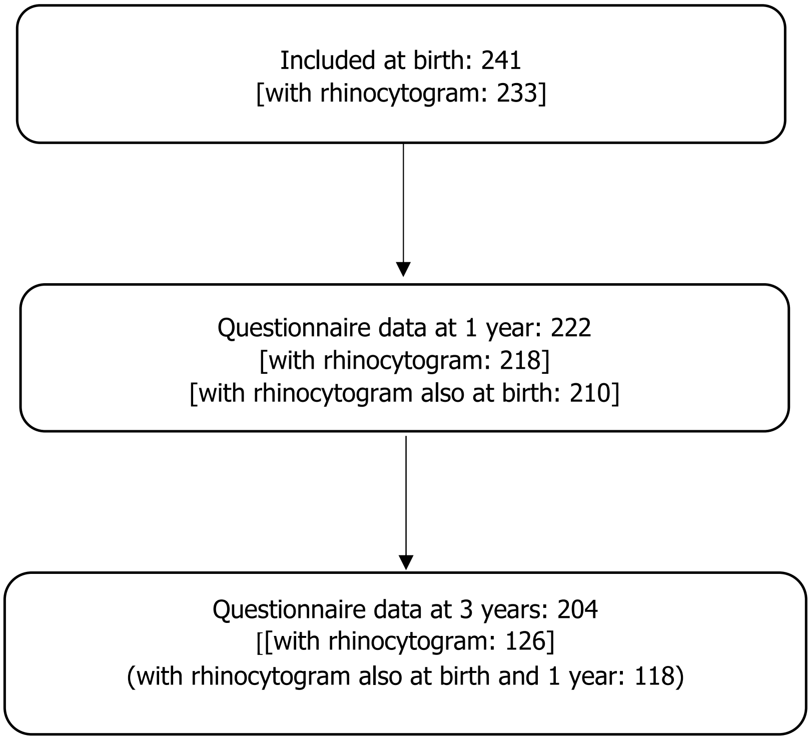

Figure S1: Patient's flow-chart

Table S1. Cross sectional association between acute otitis media (AOM), upper respiratory tract infections (URTI), bronchitis, allergy, and bronchial asthma/wheezing during the first year of life and nasal mucosal composition at 1 year.

	**AOM [n=31]**			**URTI [n=200]**			**Bronchitis [n=59]**			**Allergy [n=14]**			**Bronchial asthma/wheezing [n=25]**		
	Yes (%[a])	No (%[a])	*p*[b]	Yes (%[a])	No (%[a])	*p*	Yes (%[a])	No (%[a])	*p*	Yes (%[a])	No (%[a])	*p*	Yes (%[a])	No (%[a])	*p*
**Muciparous cells**															
**0/+**	24 (77.4)	125 (66.8)		135 (67.5)	14 (77.8)		42 (71.2)	107 (67.3)		9 (64.3)	140 (68.6)		21 (84.0)	128 (66.3)	
**++/+++/+++++**	7 (22.6)	62 (33.2)	*0.241*	65 (32.5)	4 (22.2)	*0.369*	17 (28.8)	52 (32.7)	*0.583*	5 (35.7)	64 (31.4)	*0.736*	4 (16.0)	65 (33.7)	*0.074*
**Neutrophils**															
**0/+**	7 (22.6)	59 (31.6)		60 (30.0)	6 (33.3)		14 (23.7)	52 (32.7)		0 (0)	66 (32.4)		7 (28.0)	59 (30.6)	
**++/+++/+++++**	24 (77.4)	128 (68.5)	*0.314*	140 (70.0)	12 (66.7)	*0.768*	45 (76.3)	107 (67.3)	*0.200*	14 (100)	138 (67.7)	*0.007*	18 (72.0)	134 (69.4)	*0.792*
**Eosinophils**															
**0**	29 (93.6)	174 (93.6)		185 (93.0)	18 (100)		53 (91.4)	150 (94.3)		13 (92.9)	190 (93.6)		22 (91.7)	181 (93.8)	
**+**	2 (6.5)	12 (6.5)	*0.999*	14 (7.0)	0 (0)	*0.613*	5 (8.6)	9 (5.7)	*0.432*	1 (7.1)	13 (6.4)	*0.999*	2 (8.3)	12 (6.2)	*0.657*
**Lymphocytes**															
**0**	27 (87.1)	144 (77.0)		153 (76.5)	18 (100)		46 (78.0)	125 (78.6)		8 (57.1)	163 (79.9)		21 (84.0)	50 (77.7)	
**+/++**	4 (12.9)	43 (23.0)	*0.206*	47 (23.5)	0 (0)	*0.015*	13 (22.0)	34 (21.4)	*0.917*	6 (42.9)	41 (20.1)	*0.084*	4 (16.0)	43 (22.3)	*0.473*
**Bacteria**															
**0**	14 (45.2)	77 (41.2)		85 (42.5)	6 (33.3)		24 (40.7)	67 (42.2)		8 (57.1)	83 (40.7)		12 (48.0)	79 (40.9)	
**+/++/+++/+++++**	17 (54.8)	110 (58.8)	*0.677*	115 (57.5)	12 (66.7)	*0.450*	35 (59.3)	92 (57.9)	*0.846*	6 (42.9)	121 (59.3)	*0.227*	13 (52.0)	114 (59.1)	*0.500*

[a] Column percentages.

[b] p-value from Chi-square test or Fisher exact test.
